# Remote Exercise Training Intervention During the COVID-19 Pandemic: Randomized Controlled Trial

**DOI:** 10.2196/53145

**Published:** 2024-08-08

**Authors:** Keito F A Philippi, Peter Zeier, Alexandra Brahmer, Elmo W I Neuberger, Magdalena Sandner, Matthias Hagenah, Thilo Porten, Regina Lenz, David T Ochmann, Florian Wedekink, Jörg Wischhusen, Beat Lutz, Klaus Lieb, Michèle Wessa, Perikles Simon

**Affiliations:** 1 Department of Sports Medicine, Disease Prevention and Rehabilitation Institute of Sports Science Johannes Gutenberg-University Mainz Mainz Germany; 2 Department of Clinical Psychology and Neuropsychology Institute for Psychology Johannes Gutenberg-University Mainz Mainz Germany; 3 Department of Obstetrics and Gynecology—Experimental Tumor Immunology University Clinics Würzburg Würzburg Germany; 4 Institute of Physiological Chemistry University Medical Center Johannes Gutenberg-University Mainz Mainz Germany; 5 Leibniz Institute for Resilience Research Mainz Germany; 6 Department of Psychiatry and Psychotherapy University Medical Center Johannes Gutenberg-University Mainz Mainz Germany

**Keywords:** physical exercise, web-based, COVID-19, lockdown, aerobic capacity, depression, feasibility, aerobic, anxiety, randomized controlled trial, prepandemic cohort, mental health, pandemic, remote exercise, intervention, pre-COVID-19, lockdown cohort, pandemic-related, adult, adults

## Abstract

**Background:**

Societal measures to contain the spread of COVID-19 (eg, lockdown and contact restrictions) have been associated with decreased health and well-being. A multitude of prepandemic studies identified the beneficial effects of physical exercise on both physical and mental health.

**Objective:**

We report on the feasibility of a remote physical exercise intervention and its stress-buffering potential in 2 untrained cohorts: a pre–COVID-19 cohort that completed the intervention in 2019 and a lockdown cohort that started the intervention shortly before pandemic-related restrictions were implemented.

**Methods:**

In a randomized controlled trial, participants were assigned to either an intervention group (IG; pre–COVID-19 cohort: n=7 and lockdown cohort: n=9) or a control group (CG; pre–COVID-19 cohort: n=6 and lockdown cohort: n=6). IG participants received weekly individualized training recommendations delivered via web-based support. The intervention period was initially planned for 8 weeks, which was adhered to in the pre–COVID-19 cohort (mean 8.3, SD 0.5 weeks) but was extended to an average of 17.7 (SD 2.0) weeks in the lockdown cohort. Participants’ health parameters were assessed before and after the intervention: aerobic capacity was measured as peak oxygen uptake (VO_2peak_) via cardiopulmonary exercise testing. Depressive symptoms were scored via the depression subscale of the Brief Symptom Inventory-18.

**Results:**

Dropout rates were low in both cohorts in the IG (pre–COVID-19 cohort: n=0, 0% and lockdown cohort: n=2, 16.7%) and the CG (pre–COVID-19 cohort: n=0, 0% and lockdown cohort: n=2, 20%). The mean adherence to the training sessions of the IG for both cohorts was 84% (pre–COVID-19 cohort: SD 5.5% and lockdown cohort: SD 11.6%). Aligned rank transform ANOVAs in the lockdown cohort indicated deterioration of VO_2peak_ and depressive symptoms from before to after the intervention in the CG but no longitudinal changes in the IG. Analyses in the pre–COVID-19 cohort revealed significant increases in VO_2peak_ for the IG compared to the CG (*P*=.04) but no intervention effects on depressive symptoms.

**Conclusions:**

With low dropout rates and high adherence, the remote intervention was feasible for healthy adults under regular conditions and in the face of pandemic-related stressors. Moreover, our results hint at a stress-buffering effect as well as a buffering of a lockdown-induced deconditioning of remote physical exercise interventions in the pandemic scenario, which can be used in future studies to overcome equally stressful periods of life. However, due to limited statistical power, these findings should be replicated in similar scenarios.

**Trial Registration:**

German Clinical Trials Register DRKS00018078; https://drks.de/search/en/trial/DRKS00018078

## Introduction

In March 2020, infections with SARS-CoV-2 and resulting cases of COVID-19 increased sharply in Germany and elsewhere. While the World Health Organization declared the COVID-19 outbreak as a pandemic on March 11, a first peak was reached in Germany only 3 weeks later, with about 36,000 reported cases in calendar week 14, 2020. The incidence leveled off again the following summer [1,2]. One day before the World Health Organization declared the pandemic situation, all events with over 1000 attendants were canceled in Germany. Other measures such as closing daycare centers and schools, a countrywide lockdown with restaurant closures, and extensive contact restrictions followed shortly thereafter [1].

These measures affected many people in a wide variety of situations. Even though there are findings of an increase in both physical activity and sedentary behavior in a group of university students [3], a systematic review of 64 studies with over 86,000 participants [4], however, indicates that the majority of studies [5,6] report a decrease in physical activity and an increase in sedentary behavior during lockdown periods. When addressing changes in aerobic capacity potentially induced by lockdown measures, the findings are similar. Previous studies show a decrease in aerobic capacity during the COVID-19 pandemic in different subgroups of both soccer and handball players as well as high school adolescents [7-12]. To our knowledge, there are no data regarding changes in aerobic capacity in healthy untrained adults. Aerobic capacity has been shown to be a good predictor of all-cause mortality [13-16]. Consequently, a decrease in aerobic capacity may be related to an increase in mortality rates. Improving aerobic capacity via endurance training is a possible way to counteract this deterioration [17].

Multiple studies indicated the detrimental effects of the COVID-19 pandemic on both mental and physical health. A systematic review [18] reported COVID-19–related increases in the global prevalence of major depressive disorders and anxiety disorders of 27.6% and 25.6%, respectively. Interestingly, these increases were most salient in locations with decreased human mobility. In accordance with prepandemic research [19], decreased physical activity during COVID-19 lockdown periods was significantly correlated with impaired mental health [20,21]. Conversely, physical exercise was proposed to counteract the COVID-19–related detrimental effects on mental health, as described in a systematic overview of the literature [22]. This recommendation is largely based on prepandemic research linking physical exercise to improved psychological well-being, for example, reduced levels of depression [23,24], anxiety [25], and stress reactivity [26]. Additionally, research on human resilience, that is, an individual’s ability to maintain or regain health in the face of adversity [27], revealed the stress-buffering potential of physical fitness via biological pathways, for example, optimizing hormonal stress response systems and minimizing excessive inflammation [28], and by psychological effects, for example, increasing self-efficacy [29].

Web-based interventions have been shown to be successful in increasing physical activity levels in adults when compared with waiting list control groups (CGs) [30-32]. Various studies with the same general intervention design as this study have proven to be both applicable and effective in increasing aerobic capacity in different patient groups [33-38]. Approaches that address the combination of web-based patient empowerment, as the ability of patients to positively influence their health and health behavior, as well as physical activity, show beneficial effects on patients with various chronic diseases [39].

**Figure 1:**
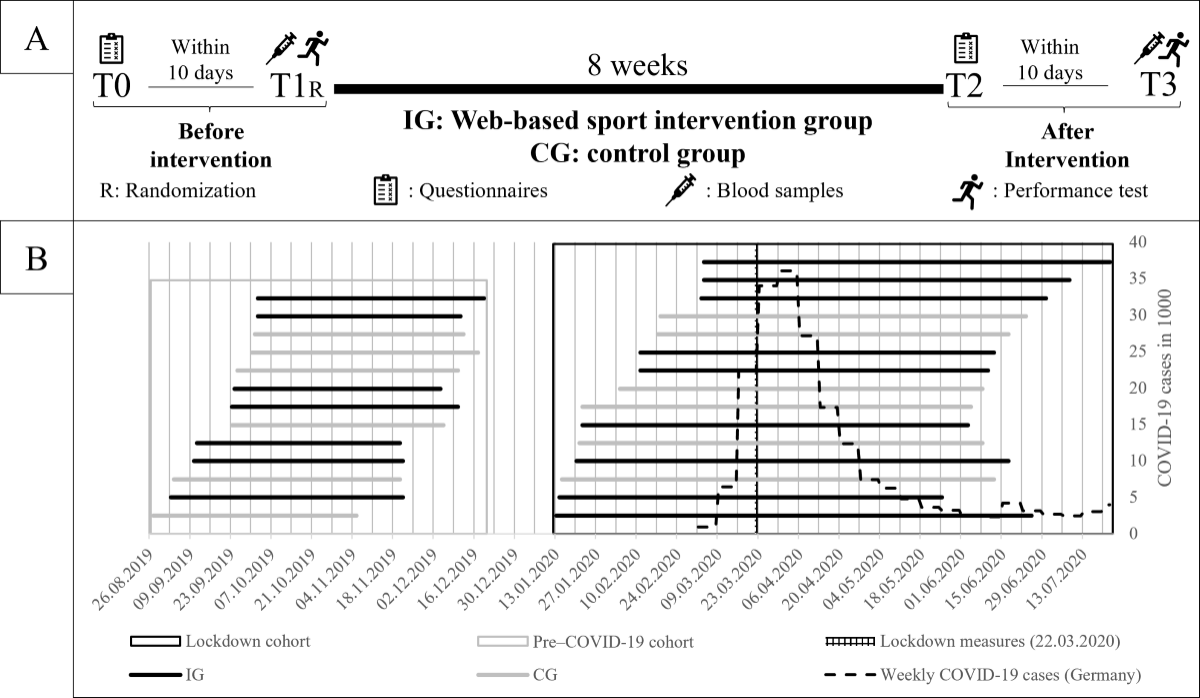
(A) Original plan for IG and CG from T0 to T3. (B) Timeline of the individual participants from T0 to T3. CG: control group; IG: intervention group.

In 2019, we started a randomized controlled trial that addressed the association of innate and acquired aerobic capacity with resilience in physically inactive healthy adults implementing remote exercise training for 8 weeks [40]. A first set of participants (pre–COVID-19 cohort; Figure 1) successfully completed the study protocol in 2019. We conducted preintervention assessments of a second set of participants (lockdown cohort) before any COVID-19–related restrictions were implemented. However, due to local restrictions, the original protocol could not have been pursued as planned in the lockdown cohort. We thus adapted the exercise intervention to investigate the stress-buffering potential of physical exercise when facing a naturalistic stressor, that is, pandemic-related restrictions. Here, we (1) report on the feasibility of a remote exercise intervention during the pandemic situation and (2) describe the impact of physical training on physical and mental health for both the pre–COVID-19 and lockdown cohorts. In accordance with previous research on physical exercise interventions, we expect a beneficial impact on both health parameters. More specifically, we hypothesize an increase in aerobic capacity (peak oxygen uptake [VO_2peak_]) and a decrease in depressive symptoms in individuals receiving remote exercise training compared to a passive CG for the pre–COVID-19 cohort. For the lockdown cohort, we hypothesize a smaller decrease in VO_2peak_ and a smaller increase in depressive symptoms in physically active individuals compared to passive controls, as the exercise training would likely counteract the detrimental effects of COVID-19–related measures, which may be caused by a more sedentary daily routine.

## Methods

### Overview

The methodology of the study was previously described in the frame of the prospective randomized controlled trial, published in a study protocol by Ochmann et al [40]. The protocol adheres to the guidelines from the SPIRIT (Standard Protocol Items: Recommendations for Interventional Trials) and the CONSORT (Consolidated Standards of Reporting Trials)–eHEALTH checklists (Multimedia Appendix 1). With the outbreak of the COVID-19 pandemic, the lockdown measures introduced in Germany in March 2020 prevented the continuation of the regular study procedure as well as further enrollment of participants. To still be able to provide sports therapy for the participants included at the beginning of 2020, the initial study protocol had to be adapted, and in the following sections, mainly these amendments are described.

### Participants and Study Design

The study design, illustrated in Figure 1A, included examinations before the intervention (T0: psychological assessments and T1: physiological assessments), an intervention period, and final examinations (T2: psychological assessments and T3: physiological assessments). Healthy untrained (less than 120 minutes per week being physically active) participants aged between 18 and 45 years were recruited via flyers distributed at the Johannes Gutenberg–University of Mainz and the University Medical Center campuses (for a detailed overview of inclusion and exclusion criteria, see Ochmann et al [40]). A total of 35 participants who provided written informed consent were randomly assigned to an intervention group (IG) or CG and completed the assessments before the intervention. This study includes data from a pre–COVID-19 cohort (2019) and a lockdown cohort (2020). Their difference in methodology relates mainly to the longer intervention period for the lockdown cohort, as shown in Figure 1B. A total of 7 participants had to be excluded from all analyses: 3 of them were excluded due to incomplete data sets and 4 participants dropped out of the study (for more details, see the *Results* section). This leads to a final sample of 13 participants in the pre–COVID-19 cohort (IG: n=7 and CG: n=6) and 15 participants in the lockdown cohort (IG: n=9 and CG: n=6). Further descriptions of the final study population can be found in the *Results* section.

### Training Intervention

Weekly training in the IG consisted of progressive continuous and interval runs with the goal of gradually increasing aerobic capacity (for details, see Ochmann et al [40]). The trainer individually adapted the training to the weekly feedback provided by the participant via web-based support. As we did not receive any reports regarding major technical problems from the study participants, we assume that the technical use of web-based support was not a challenge from the perspective of the test participants. However, if more intensive clarification was required, a telephone consultation was arranged. After verifying that the participant complied with the training recommendations, the 2 factors of perceived ailment and load mainly determined the adjustments. Since the original training progression had been designed for an 8-week study period, this design could not cover an indefinite period. Therefore, the trainer mainly focused on the continuation of the participants’ sporting activities. As originally planned, the principle of training adjustment was also applied to the lockdown cohort. Thus, depending on the perceived ailment and load, the training was reduced, maintained, or intensified.

### Measures

The German version of the Brief Symptom Inventory-18 (BSI-18) [41,42] was applied to measure psychological distress. The BSI-18 contains 3 subscales, that is, depression, anxiety, and somatization, which are assessed as sum scores of 6 items each, as well as a Global Severity Index calculated as a sum across all 18 items. All participants worked twice on the BSI-18, once before the intervention period (T0) and once after the period (T2). In accordance with our hypotheses, we limited our main analyses to the depression subscale of the BSI-18.

VO_2peak_ was determined as a main indicator of aerobic capacity. It was chosen over VO_2max_ as the main parameter since we did not expect the occurrence of a plateau in testing untrained participants [43]. VO_2peak_ was measured during a stepwise incremental running test starting at 4 km/h and increasing velocity every 3 minutes by 1.5 km/h (for further details, see the initial study protocol by Ochmann et al [40]). The determination took place once before the intervention period (T1) and once after the period (T3). Training adherence was calculated by dividing documented training sessions by recommended training sessions.

### Test for SARS-CoV-2 Seroconversion

Participants in the lockdown cohort were tested for SARS-CoV-2 seroconversion to exclude an undetected infection via the enzyme-linked immunosorbent assay described by Notz et al [44]. Venous blood was drawn in tripotassium-ethylenediaminetetraacetic acid–covered Monovettes (Sarstedt) and was processed within 5 minutes after blood collection. Platelet-free plasma was prepared by 2 rounds of centrifugation for 15 minutes at 2500×g and room temperature. Plasma was aliquoted and stored at –80 °C until enzyme-linked immunosorbent assay measurement.

At T1, all participants showed no sign of seroconversion, as expected prior to the pandemic situation. At T3, seroconversion of 2 participants, who had already stated a SARS-CoV-2 infection during the intervention phase, was confirmed. Medical anamnesis as well as pulmonary function and spiroergometric tests ensured that these participants did not experience any aftermaths affecting their ability to participate in the study. Besides this, no seroconversions or stated SARS-CoV-2 infections were reported. Therefore, no participants were excluded due to the SARS-CoV-2 infection.

### Statistical Analyses

SPSS (version 23; IBM Corp) was used for data analysis. Aligned rank transform (ART) ANOVAs [45] were conducted for each cohort separately to test our hypotheses, with time (before vs after the intervention) as a within-subject factor, group (IG vs CG) as a between-subject factor, and VO_2peak_ and depression scores as dependent variables. Follow-up Wilcoxon tests were performed to contrast specific time- and group-related effects.

### Ethical Considerations

The study protocol follows the standards of the Declaration of Helsinki of the World Medical Association, and ethics approval was obtained by the medical association Rhineland-Palatinate (July 29, 2019; IRB approval 2019-14305). The original study was registered with the German Clinical Trials Register (DRKS00018078; October 2, 2019). All participants provided informed consent before participation and were informed about the ability to opt out of the study at any time. Furthermore, all data were pseudonymized. As compensation for complete participation, 15 smartwatches (M430, Polar Electro Oy) were raffled among all participants.

## Results

### Study Population and Feasibility of the Randomized Controlled Exercise Intervention

Due to the unexpected occurrence of the COVID-19 pandemic and its related restrictions, we have the unique opportunity to report on the feasibility of the conduction of a randomized controlled remote exercise intervention under these circumstances. Whereas a pre–COVID-19 cohort was able to proceed through the study protocol as planned, the intervention period and the final examinations of the lockdown cohort could not be conducted as planned. On March 16, 2020, all participants were notified about an indefinite extension of the intervention period. The local restrictions, in the form of the first lockdown, in Germany started on March 27, 2020. Figure 1B shows all participant timelines combined with a visualization of the weekly COVID-19 cases. Table 1 provides additional measurements describing the study population. Participants in the IG and CG did not show significant differences regarding VO_2peak_ (pre–COVID-19 cohort: *P*=.22 and lockdown cohort: *P*=.52) or depression scores (pre–COVID-19 cohort: *P*=.43 and lockdown cohort: *P*=.70) before the intervention.

**Table 1:** Description of the study population (N=28).

Characteristics			Pre–COVID-19 cohort							Lockdown cohort							Total
			CG^a^		IG^b^		Subtotal		CG			IG		Subtotal			
**Sex, n (%)**																	
	Female	3 (11)		5 (18)		8 (29)		4 (14)			5 (18)		9 (32)		17 (61)		
	Male	3 (11)		2 (7)		5 (18)		2 (7)			4 (14)		6 (21)		11 (39)		
Age (years), mean (SD)			26.5 (9.5)		25.6 (3.9)		26.0 (6.7)		26.7 (4.6)			25.4 (5.9)		25.9 (5.3)		26.0 (5.9)	
Weight (kg), mean (SD)			68.4 (13.2)		75.7 (11.1)		72.4 (12.2)		68.6 (8.4)			69.3 (9.5)		69.0 (8.8)		70.6 (10.4)	
Height (cm), mean (SD)			172.2 (9.9)		173.9 (12.4)		173.1 (10.9)		170.0 (7.6)			175.4 (7.1)		173.3 (7.6)		173.2 (9.1)	
BMI (kg/m^2^), mean (SD)			23.2 (4.2)		25.0 (2.8)		24.2 (3.5)		23.9 (3.9)			22.5 (2.3)		23.0 (3.0)		23.6 (3.2)	
Body fat (%), mean (SD)			21.5 (9.2)		26.7 (9.9)		24.3 (9.5)		22.0 (11.1)			18.0 (7.1)		19.6 (9.0)		21.8 (9.4)	
Muscle mass (kg), mean (SD)			49.7 (7.9)		51.8 (9.1)		50.8 (8.3)		49.5 (3.7)			53.3 (8.9)		51.8 (7.3)		51.3 (7.7)	
VO_2peak_^c^ (mL/min/kg), mean (SD)			40.4 (6.2)		35.0 (8.4)		37.5 (7.7)		37.6 (7.8)			39.9 (6.1)		39.0 (6.7)		38.3 (7.1)	
Speed at IAT^d^ (km/h), mean (SD)			7.8 (1.5)		7.7 (2.3)		7.7 (1.9)		6.7 (1.2)			8.5 (2.1)		7.8 (2.0)		7.8 (1.9)	
Maximum heart rate (per minute), mean (SD)			192.8 (8.0)		196.0 (12.4)		194.5 (10.3)		193.3 (5.4)			201.0 (7.6)		197.9 (7.6)		196.4 (9.0)	
BSI^e^—depression, mean (SD)			2.5 (1.2)		3.7 (3.4)		3.2 (2.6)		1.8 (1.7)			1.6 (1.0)		1.7 (1.3)		2.4 (2.1)	
Total intervention weeks, mean (SD)			8.3 (0.5)		8.3 (0.5)		8.3 (0.5)		17.3 (1.0)			17.7 (2.0)		17.5 (1.6)		13.3 (4.8)	
Adherence (%), mean (SD)			N/A^f^		84.0 (5.5)		N/A		N/A			84.0 (11.6)		N/A		84.0 (9.2)	
Weekly training sessions, mean (SD)			N/A		2.3 (0.4)		N/A		N/A			2.3 (0.6)		N/A		2.3 (0.5)	
Total training sessions, mean (SD)			N/A		18.6 (2.9)		N/A		N/A			41.3 (12.9)		N/A		31.4 (15.1)	

^a^CG: control group.

^b^IG: intervention group.

^c^VO_2peak_: peak oxygen uptake.

^d^IAT: individual anaerobic threshold.

^e^BSI: Brief Symptom Inventory.

^f^N/A: not applicable.

The duration of the intervention for the lockdown cohort between T1 and T2 increased on average to 17.3 (SD 1.0) weeks for the CG and 17.7 (SD 2.0) weeks for the IG. The intervention times for the pre–COVID-19 cohort in comparison lasted a mean of 8.3 (SD 0.5) weeks for the CG and 8.3 (SD 0.5) weeks for the IG. The mean number of training sessions per week, on the other hand, showed no difference (pre–COVID-19 cohort: mean 2.3, SD 0.4 and lockdown cohort: mean 2.3, SD 0.6). However, in the lockdown cohort, the training became more individualized the longer the training intervention lasted. While the framework of the original training progression was retained, the structure of the training sessions was adjusted to the individual demands. This led to a slightly higher effort for the trainer without increasing communication with the participants. For example, there were individuals who preferred to maintain a frequency of 3 units per week during the lockdown, while others preferred 5 units per week. Furthermore, in addition to endurance runs and interval units, combinations of moderate and vigorous intensities as well as Fartlek runs (individual pace and intensity variations during unstructured interval training) were included. Depending on how the participants accepted these training methods, they were recommended more or less frequently. However, the overall weekly volume was always maintained. Finally, the mean adherence, that is, the quotient of training sessions performed and recommended, did not differ between the cohorts (84% for both cohorts) but differed in the SD (pre–COVID-19 cohort: 5.5% and lockdown cohort: 11.6%; Table 1).

There were low dropout rates in both cohorts in the IG (pre–COVID-19 cohort: n=0, 0% and lockdown cohort: n=2, 16.7%) and the CG (pre–COVID-19 cohort: n=0, 0% and lockdown cohort: n=2, 20%). No participants in the pre–COVID-19 cohort and 4 participants in the lockdown cohort dropped out from the study. However, there was no study dropout specifically induced by the prolongation of the intervention period. In total, 2 participants dropped out of the CG due to a change of personal situation, and 2 participants dropped out of the IG due to medical reasons not related to the study or COVID-19. One of the latter participants dropped out 16 weeks after T1, and the other dropped out 4 weeks after T1 and before the lockdown started.

Taken together, the adaptation of the training intervention to the pandemic situation was well feasible, and the training adherence as well as the dropout rate were adequate. Despite the small sample sizes induced by introducing a pre–COVID-19 cohort and a lockdown cohort, the study populations in the IG and the CG for both cohorts were highly similar.

### Effects of Exercise Intervention on VO2peak and Depression

Since the conduction of our remote physical exercise intervention turned out feasible and the study population was highly comparable at T1, specifically regarding VO_2peak_ and depression scores, we investigated a potentially beneficial effect of the physical exercise intervention on both health variables in the next step. Since the interventions in the pre–COVID-19 and lockdown cohorts differed especially regarding training durations, we analyzed both cohorts separately. We performed nonparametric analyses to account for the small sample sizes in both cohorts. More specifically, ART [45] was conducted to investigate the main and interaction effects of group (intervention vs control) and time (before vs after the intervention) on depression and VO_2peak_. Follow-up 1-sample Wilcoxon signed rank tests were performed to investigate changes in both health parameters within groups.

**Figure 2:**
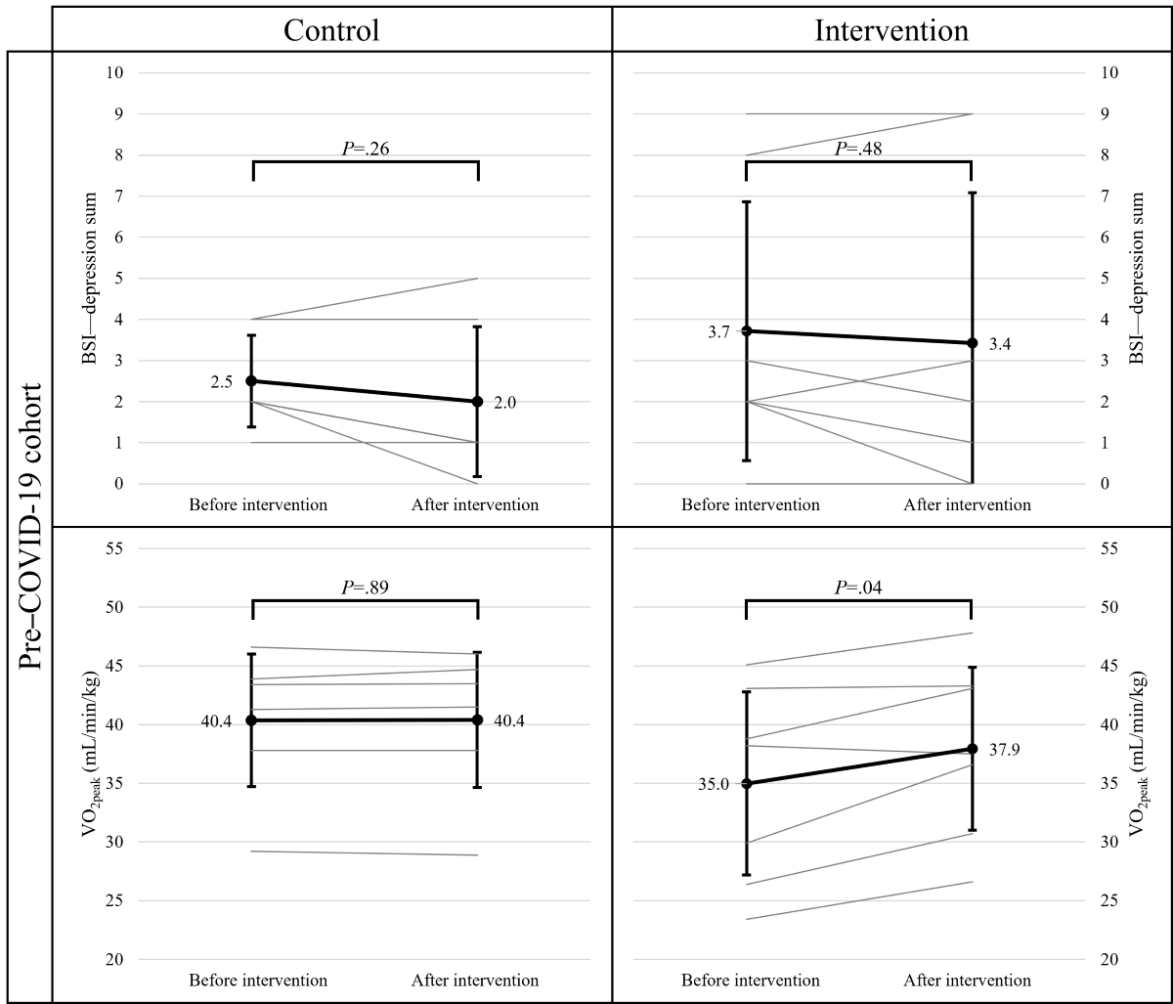
Results of Wilcoxon tests before and after the intervention for depression and VO_2peak_ in the pre–COVID-19 cohort. BSI: Brief Symptom Inventory; VO_2peak_: peak oxygen uptake.

Regarding the pre–COVID-19 cohort, the ART ANOVA with VO_2peak_ as a dependent variable revealed nonsignificant main effects of time (*F*_1,11_=0.98; *P*=.34; η^2^=0.08) and group (*F*_1,11_=0.01; *P*=.92; η^2^<0.01) but a significant time×group interaction (*F*_1,11_=5.27; *P*=.04; η^2^=0.32). However, a second ART ANOVA on depression did not indicate significant main effects of time (*F*_1,11_=0.45; *P*=.52; η^2^=0.04) and group (*F*_1,11_=0.18; *P*=.68; η^2^=0.02) nor a significant time×group interaction effect (*F*_1,11_=0.82; *P*=.39; η^2^=0.07). One-sample Wilcoxon tests for each group separately indicated a significant increase in VO_2peak_ (before the intervention: mean 34.99, SD 8.43 and after the intervention: mean 37.94, SD 7.48; *P*=.04) in the IG (Figure 2).

**Figure 3:**
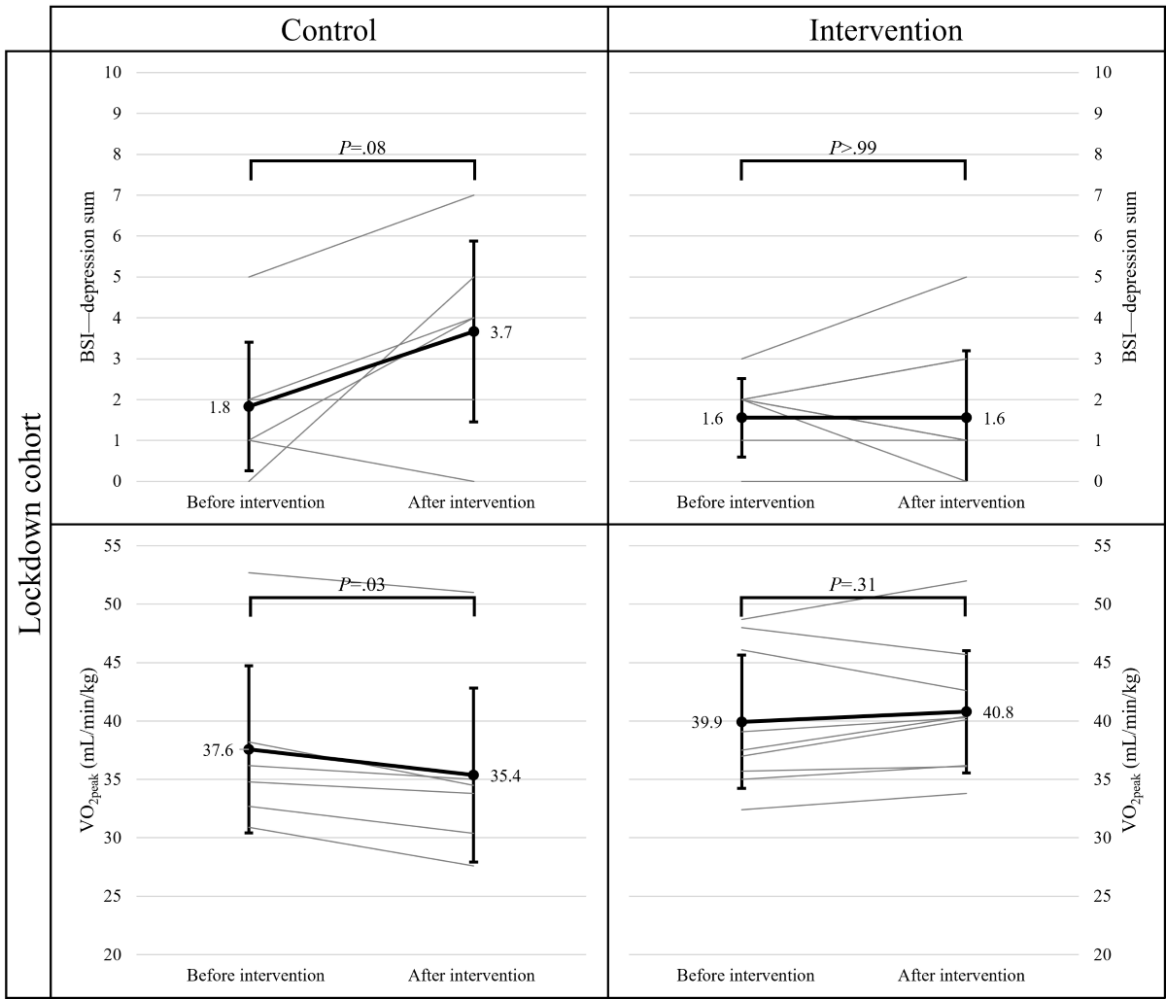
Results of Wilcoxon tests before and after the intervention for depression and VO_2peak_ in the lockdown cohort. BSI: Brief Symptom Inventory; VO_2peak_: peak oxygen uptake.

Regarding the lockdown sample, the ART ANOVAs revealed significant time×group interaction effects on VO_2peak_ (*F*_1,13_=11.44; *P*=.01; η^2^=0.47) and a marginally significant interaction effect on depression (*F*_1,13_=3.90; *P*=.07; η^2^=0.23). The ART ANOVAs revealed no significant main effects of time or group on VO_2peak_ (time: *F*_1,13_=0.04; *P*=.85; η^2^<0.01 and group: *F*_1,13_=0.09; *P*=.77; η^2^=0.01) or depression scores (time: *F*_1,13_<0.01; *P*=.98; η^2^<0.01 and group: *F*_1,13_=0.23; *P*=.64; η^2^=0.02). As indicated in Figure 3, follow-up Wilcoxon tests indicated a significant decrease of VO_2peak_ (before the intervention: mean 37.58, SD 7.84 and after the intervention: mean 35.38, SD 8.16; *P*=.03) and a marginally significant increase in depression (before the intervention: mean 1.83, SD 1.72 and after the intervention: mean 3.67, SD 2.42; *P*=.08) in the CG.

Taken together, our findings indicate that while participants in the CG experienced a decrease in aerobic capacity and an increase in depression levels during the first COVID-19–induced lockdown, participants in the IG remained stable on both parameters. On the other hand, under normal conditions (pre–COVID-19 cohort), only the aerobic capacity increased significantly in the IG.

## Discussion

### Principal Findings

In this study, we had the unique chance to investigate the feasibility of a remote physical exercise intervention and, furthermore, its potentially beneficial effect on physical and mental health during a stress-inducing COVID-19 lockdown situation. With a low dropout rate and high adherence to the training sessions, the intervention was well feasible. Moreover, our results hint at the stress-buffering potential of the exercise intervention, although the statistical power was limited due to the small sample size.

The continuation of our study was well feasible in the prevailing pandemic situation mainly due to the use of a web-based training intervention. All training support structures implemented in this study procedure could be maintained as planned, and high adherence values and low dropout rates in the lockdown cohort were achieved. Most importantly, there was no need for physical contact, while the continuation of a program including such an approach would not have been possible. The training could simply continue as before since the trainer and participant were in contact exclusively via digital means. This remote contact allowed for fast support in case of problems, as there was no need to wait for the next training session. Changes that were necessary to keep the intervention running included the extension of the training plans from originally 8 weeks to a longer, undefined period, stronger exchange between participant and trainer, and more individualized training plans. Heart rate and other parameters were tracked continuously, and the trainer had a very detailed insight into the physiological variables during the workout, which facilitated personalization processes. Moreover, the individuals’ training preferences and personal motivation could be easily considered through frequent feedback via chat messages and training protocols completed by the participants. However, this increased the trainer’s workload to a certain level; but in interventions that keep contact with the participants exclusively via remote, it is important for the trainer to follow a certain regularity, provide frequent feedback, and engage with the participant on a personal level with genuine interest anyway [46]. Finally, the adaptations were feasible, and we had the opportunity to further evaluate the effect of our physical exercise intervention on physical and mental health.

Next, we investigated the stress-buffering potential of our remotely supervised physical exercise intervention facing a severely restrictive life situation in terms of daily activity and social interactions. Specifically, we were interested in the potentially beneficial effects of regular physical exercise on both VO_2peak_ and depressive symptoms in the IGs. The results of the pre–COVID-19 cohort showed the expected significant increase in aerobic capacity for the IG but no changes in the CG. The increase in VO_2peak_ in the healthy pre–COVID-19 cohort is similar to changes in patient cohorts with different diseases in comparable intervention studies [35,37]. However, the exercise intervention did not significantly decrease depression scores, most likely due to only minimal depressive symptoms before intervention. In the lockdown cohort, time×group interactions revealed a significant deterioration of VO_2peak_ and a marginally significant decrease of depression in the CG but not in the IG. While the results in the pre–COVID-19 cohort indicate the overall effectiveness of the remote training intervention, the IG of the lockdown cohort maintained their physical and mental health in the face of a naturalistic stressor, that is, the pandemic-related lockdown. The latter finding is in line with previous studies on the stress-buffering effects of physical exercise [28,29]. Accordingly, the training intervention has likely compensated for the detrimental effects of a sedentary routine due to COVID-19–related restrictions.

### Comparison to Prior Work

Our findings corroborate the stress-buffering potential of physical exercise previously indicated by multiple cross-sectional studies [47]. However, only a limited number of randomized controlled trials, especially in real-life stressor settings, have been conducted so far. For example, 20 weeks of aerobic exercise training were shown to have beneficial effects on the emotional stress reactivity of healthy students [48] for the prevention of stress-related mental health impairments. In line with the current findings, future studies should focus on real-life stressors when investigating the stress-buffering potential of physical exercise interventions.

The results hint at a stress-buffering effect of physical exercise after ~18 weeks. It remains unclear whether fewer training sessions, for example, 8 weeks, as originally planned, would yield similar stress-buffering effects. Most studies on physical exercise use intervention periods of 8-12 weeks, whereas a meta-analysis by Bruner et al [48] shows stronger effects for different factors of positive youth development in studies exceeding 10-week intervention periods. Interventional studies with overfat individuals show the most beneficial effects on cardiorespiratory characteristics in interventions that last at least 12 weeks [49]. The longer intervention period may thus also have enhanced effects on aerobic capacity. Further randomized controlled trials should define the modes, volumes, and lengths of physical exercise that elicit the stress-buffering effect of routine physical exercise.

### Strengths and Limitations

To our knowledge, this is the first study to investigate the benefits of a training intervention at the onset of the COVID-19 pandemic in healthy untrained participants, which measures aerobic capacity by VO_2peak_. Specifically, participants started the intervention shortly before pandemic-related restrictions were implemented. The significant improvements in VO_2peak_ observed in the pre–COVID-19 cohort were not achieved under lockdown conditions. Instead, aerobic capacity in the IG of the lockdown cohort did not change significantly, while it decreased significantly in the CG. In line with findings from similar studies, participants in the CG of the lockdown cohort may have experienced detraining or deconditioning. Ammar et al [50] showed a detraining effect after an initial 8-week fitness-dance training in older adults with mild cognitive impairment. While detraining in general is defined as the partial or complete loss of training-induced adaptations [51], our observations show a similar effect even without initial training in untrained participants. This deconditioning effect is most likely induced by the overall lowered physical activity [52-54], which different studies show during the COVID-19 lockdowns [4,6]. To conclude, training interventions may stop or at least buffer lockdown-induced deconditioning even in untrained healthy participants.

However, as external circumstances introduced unforeseen differences between the first and second cohorts, small sample sizes limited the robustness of our findings, which should consequently be interpreted with caution. While the results indicate large effect sizes regarding the intervention on aerobic capacity in the pre–COVID-19 cohort (η^2^=0.32) and on both health parameters in the lockdown cohort (VO_2peak_: η^2^=0.47 and depression: η^2^=0.23), a follow-up study in a more representative sample would enable a more conclusive interpretation of these findings. Along these lines, the limited statistical power did not permit analyses of potential mediator variables. Future studies in larger samples should analyze resilience mechanisms affected by the training intervention, for example, improved hypothalamic-pituitary-adrenal axis activity or increased self-efficacy. Still, the coincidental (or serendipitous) nature of our findings renders an equivalent replication study rather impossible. Nevertheless, a follow-up study may apply a somewhat similar design by implementing the training intervention in a population that experiences a more predictable real-life stressor with similar characteristics, for example, increased sedentary behavior and less social interactions. A population of interest may, for example, consist of students facing an examination period.

### Conclusions

The outbreak of the COVID-19 pandemic and the implemented lockdown periods led to an increase in sedentary behavior associated with decreased health and well-being. We had the unique opportunity to investigate the feasibility and the stress-buffering potential of a remote physical exercise intervention with healthy untrained participants under these circumstances. Our results indicate that a lockdown cohort, which started a remotely supervised physical exercise intervention shortly before pandemic-related restrictions, significantly benefited from regular physical exercise in terms of aerobic capacity and depressive symptoms. Low dropout rates and high adherence to the training, despite the prevailing lockdown-related restrictions, underscore the feasibility and importance of remote physical exercise training approaches in such scenarios. Furthermore, our findings highlight the importance of physical exercise to cope with stressful life situations and its integration into daily life, for example, on a personal level or via occupational health management. Future randomized controlled trials regarding further relevant real-life stressors may reveal the broad stress-buffering potential of physical exercise.

## Appendix

Multimedia Appendix 1 CONSORT (Consolidated Standards of Reporting Trials)-EHEALTH V1.6 checklist.

## Abbreviations

ART: aligned rank transform
BSI-18: Brief Symptom Inventory-18
CG: control group
CONSORT: Consolidated Standards of Reporting Trials
IG: intervention group
SPIRIT: Standard Protocol Items: Recommendations for Interventional Trials
VO_2peak_: peak oxygen uptake
